# [^18^F]FET-βAG-TOCA: The Design, Evaluation and Clinical Translation of a Fluorinated Octreotide

**DOI:** 10.3390/cancers12040865

**Published:** 2020-04-02

**Authors:** Louis Allott, Suraiya Dubash, Eric O. Aboagye

**Affiliations:** Comprehensive Cancer Imaging Centre, Imperial College London, Hammersmith Hospital, Du Cane Road, London W12 0NN, UK; l.allott@imperial.ac.uk (L.A.); s.dubash@imperial.ac.uk (S.D.)

**Keywords:** fluorine-18, 2-[^18^F]fluoroethylazide, radiopharmaceuticals, clinical translation, positron emission tomography, octreotide, Lutathera

## Abstract

The success of Lutathera™ ([^177^Lu]Lu-DOTA-TATE) in the NETTER-1 clinical trial as a peptide receptor radionuclide therapy (PRRT) for somatostatin receptor expressing (SSTR) neuroendocrine tumours (NET) is likely to increase the demand for patient stratification by positron emission tomography (PET). The current gold standard of gallium-68 radiolabelled somatostatin analogues (e.g., [^68^Ga]Ga-DOTA-TATE) works effectively, but access is constrained by the limited availability and scalability of gallium-68 radiopharmaceutical production. The aim of this review is three-fold: firstly, we discuss the peptide library design, biological evaluation and clinical translation of [^18^F]fluoroethyltriazole-βAG-TOCA ([^18^F]FET-βAG-TOCA), our fluorine-18 radiolabelled octreotide; secondly, to exemplify the potential of the 2-[^18^F]fluoroethylazide prosthetic group and copper-catalysed azide-alkyne cycloaddition (CuAAC) chemistry in accessing good manufacturing practice (GMP) compatible radiopharmaceuticals; thirdly, we aim to illustrate a framework for the translation of similarly radiolabelled peptides, in which in vivo pharmacokinetics drives candidate selection, supported by robust radiochemistry methodology and a route to GMP production. It is hoped that this review will continue to inspire the development and translation of fluorine-18 radiolabelled peptides into clinical studies for the benefit of patients.

## 1. Introduction

The field of nuclear medicine has advocated a theragnostic approach towards personalised medicine by combining patient stratification with positron emission tomography (PET) imaging and radionuclide therapy using theragnostic pairs such as gallium-68 (^68^Ga, β^+^ emitter) and lutetium-177 (^177^Lu, β^−^ emitter) [1]. This approach was exemplified for neuroendocrine tumours (NETs) expressing the somatostatin receptor (SSTR) by combining PET imaging with [^68^Ga]Ga-DOTA-TATE (NETSPOT™ in the US) or [^68^Ga]Ga-DOTA-TOC (SomakitTOC™ in Europe) to select patients to receive the molecularly targeted radionuclide therapeutic, Lutathera™ ([^177^Lu]Lu-DOTA-TATE). The NETTER-1 trial has shown promising results for the treatment of somatostatin receptor type 2 (SSTR2) NETS with Luthathera™; approval by the Food and Drug Administration (FDA) and European Medicines Agency (EMA) demonstrates the first implementation of personalised nuclear medicine that is likely to become routine [2,3,4].

A bottleneck in the routine treatment of patients using Lutathera™ is expected to result from the low throughput (compared to fluorine-18 radiopharmaceuticals) of [^68^Ga]Ga-DOTA-TATE PET imaging for stratifying patients. Several factors result in [^68^Ga]Ga-DOTA-TATE being unable to satisfy high clinical demand: 1) the relatively low radioactivity of gallium-68 (^68^Ga) produced by ^68^Ge/^68^Ga generators (typically 1.8 GBq when new, from a generator authorized for patient use) limits the number of clinical doses produced from a single elution; 2) the half-life (t_1/2_ = 68 min) is incompatible with the satellite distribution model (as used for the production and distribution of [^18^F]FDG) where production sites distribute radiopharmaceuticals to remote imaging facilities; 3) accessing new ^68^Ge/^68^Ga generators is problematic as the worldwide supply is limited [5]. Despite limitations, gallium-68 remains a useful and attractive radioisotope for the development of new radiopharmaceuticals. The simplicity of metal-based radiochemistry with rapid, mild reaction conditions negate prosthetic and protecting group strategies, allowing for the swift evaluation of radioconjugates in pre-clinical models [6,7]. The generator produced radioisotope is ideal for research institutions and hospital radiopharmacies as on-demand radioactive doses are straightforward to access and for some, more convenient and inexpensive than fluorine-18 (^18^F, t_1/2_ = 110 min). Furthermore, the progression of ^68^Ga-radiopharmaceuticals into phase I and II human studies can be accommodated by ^68^Ge/^68^Ga generator produced radioisotope as demand is usually limited to 1–2 patients per generator elution, perhaps twice per day [8]. The technological leap between the early clinical evaluation of ^68^Ga-radiopharmaceuticals and routine practice is where many foresee challenges; the uninterrupted access to PET radiopharmaceuticals is of paramount importance when patients require stratification to access ground-breaking therapies such as Lutathera™. Cyclotron produced gallium-68 may be able to increase the throughput of scans at institutions with on-site cyclotrons, but it remains to be seen if the centralised production of ^68^Ga-radiopharmaceuticals and distribution to satellite imaging facilities is a viable model. Cyclotron-produced ^68^Ga is challenging to access in a form suitable for radiolabeling, especially compared to the simplicity of generator-produced radioisotope; radionuclidic impurities (i.e., ^67^Ga t_1/2_ = 78 h) are also a concern [9].

**Figure 1:**
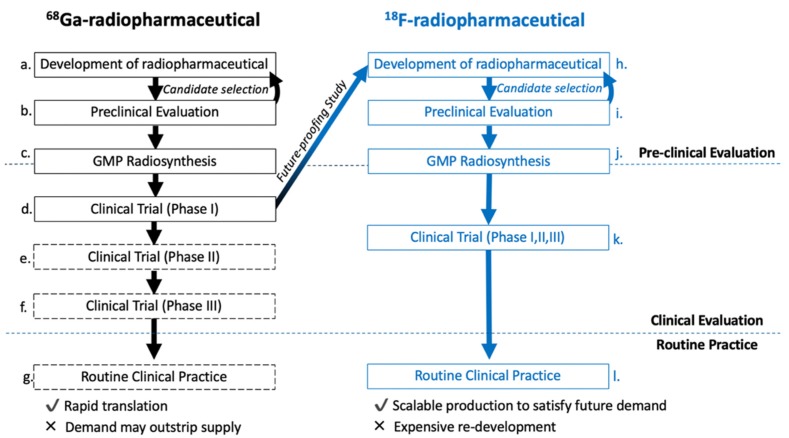
Proposed workflow for the development of gallium-68 radiopharmaceuticals alongside complementary fluorine-18 analogues.

The development pipeline for ^68^Ga-radiopharmaceuticals should be restructured. Translational scientists must think beyond the requirements of supporting early phase (I–II) clinical trials and keep in mind the possibility of entering routine clinical practice with increasing demand over time. The development and evaluation of copper-64 radiolabelled DOTATATE ([^64^Cu]Cu-DOTA-TATE) was hypothesised to improve the logistics of patient stratification by PET, due to the long half-life of the isotope (t_1/2_ = 12.7 h) [10,11,12]. Head-to-head evaluation of [^64^Cu]Cu-DOTA-TATE with [^111^In]In-DTPA-octreotide and [^68^Ga]Ga-DOTA-TOC showed superior performance of [^64^Cu]Cu-DOTA-TATE which showcased the radioconjugate as a sensitive and convenient SSTR2 radiopharmaceutical for routine use [13]. Despite promising results, the production of copper-64 is the preserve of specialist PET centres and the radioisotope itself does not have favourable decay characteristics for PET imaging (β^+^_em_ = 17.8%, β^−^_em_ = 38.4%) [14]. We propose a pipeline that exemplifies the development of ^68^Ga-radiopharmaceuticals alongside complementary fluorine-18 analogues, that can be produced in an almost limitless quantity and widespread availability (Figure 1); the decay characteristics of fluorine-18 (t_1/2_ = 110 min, β^+^_em_ = 97%) are ideal for same-day radiopharmaceutical manufacture, transportation and PET imaging. Cyclotron-produced fluorine-18 is the workhorse of modern PET with thousands of cyclotrons in Europe and the US each producing nucleophilic fluoride (^18^F^−^) in vast quantities (up to 700 GBq) on a daily basis for the radiosynthesis of [^18^F]FDG [15]. The demand for SSTR2 imaging is unlikely to exceed that of FDG-PET, but fluorine-18 certainly provides the bandwidth necessary for potential growth.

We propose that “future-proofing” studies, where fluorine-18 analogues of ^68^Ga-radiopharmaceuticals under investigation are developed right after phase I clinical trials. The development of new fluorine-18 radioconjugates may seem laborious after progressing gallium-68 candidates into clinical trials, but it provides an ideal opportunity to improve access to radiopharmaceuticals for the benefit of patients. Fluorine-18 derivatives of desirable radioconjugates not only improve because of better production scalability and therefore accessibility, but optimization of their structure to incorporate a fluorine-18 radiolabelled appendage can also improve tumour uptake and pharmacokinetics (PK), as demonstrated later in this review. The question of radiopharmaceuticals meeting clinical demand is not only limited to gallium-68 but should also encompass the development of any radiochemistry methodology. Scalability is important for routine clinical practice and therefore robust production strategies are key to satisfying future demand and accessibility.

With this in mind, we and others have followed a similar pipeline as shown in Figure 1, and developed fluorine-18 radiolabelled somatostatin analogues (SSA) as an alternative to [^68^Ga]Ga-DOTA-TATE by investigating a plethora of ^18^F-labeling strategies and structural modifications [16]. In this review, we aim to summarize the field of fluorine-18 radiolabelled somatostatin analogues (^18^F-SSA) with particular focus on the bench-to-bedside development of our radiopharmaceutical [^18^F]fluoroethyltriazole-βAG-TOCA ([^18^F]FET-βAG-TOCA), highlighting important considerations for translating radiolabelled peptides. By sharing our experience and development pipeline, we hope to provide a foundation of knowledge that may assist the PET community in progressing radiolabelled peptides into the clinic [17].

## 2. Fluorine-18 Radiolabelled Somatostatin Analogues

**Figure 2:**
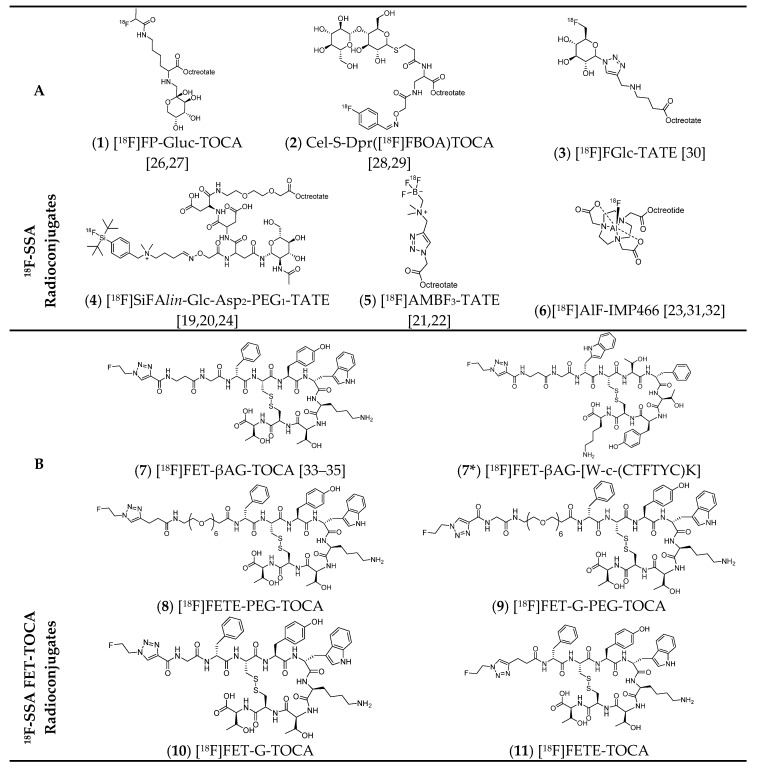
Fluorine-18 radiolabelled somatostatin analogues: (**A**) examples of a variety of ^18^F-SSA radioconjugates; (**B**) a focused ^18^F-SSA library based on FET-TOCA. Compound 7* is a scrambled derivative of **7** and was used as a negative control.

Several promising ^18^F-SSA targeting SSTR2 are reported in the literature, differing in their radiolabelling and linker chemistry but are all based around the octreotate/octreotide peptide pharmacophore (Figure 2). Early attempts to radiolabel somatostatin analogues with lipophilic aromatic ^18^F-prosthetic groups resulted in poor tumour uptake and high liver accumulation [18]. To compensate for the increased lipophilicity of these prosthetic group approaches, radioconjugates were synthesised containing hydrophilic moieties to improve target specificity and biodistribution. As a result, many examples of ^18^F-SSA radioconjugates contain single (compounds **1**, **3** and **4**) or multiple (compound **2**) hydrophilic carbohydrate moieties to reduce the lipophilicity of the radioconjugate. Similarly, charged moieties (compound **4** and **5**) were also included to increase hydrophilicity (Figure 2, Table 1). To avoid the long radiosynthesis times and multi-step reactions needed to access some ^18^F-SSA probes, alternative radiolabelling strategies to conventional ^18^F-C bond formation were exploited for their rapid procedures. These included the implementation of radiochemistry utilising ^18^F-silicon (**4**) [19,20], ^18^F-boron (**5**) [21,22] and ^18^F-aluminium (**6**) [23] bonds which can be accessed without HPLC purification of the final radioconjugate [24]. A comprehensive review on these ^18^F-SSA is reported in the recent literature [16]. In addition, SSTR2 antagonists (e.g., JR11 and BASS) are beyond the scope of this review but may be an important development for imaging and therapy in the future [25].

## 3. Strategic Aim

**Figure 3:**
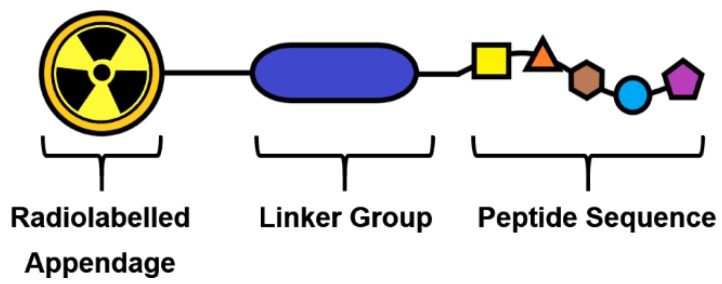
Generic representation of a three-component peptide radioconjugate.

The structure of ^18^F-SSA peptides is typically based around three components (Figure 3). Firstly, the peptide sequence with a high affinity and selectivity for the SSTR2 receptor is selected, then a radiolabelled appendage to support fluorine-18 radiochemistry is rationalised together with a linker strategy to permit radiolabelling via the chosen prosthetic group strategy and to modulate the PK of the radioconjugate.

Identifying the strengths and weaknesses of existing ^18^F-SSA probes reported in the literature allowed key aims and design criteria to be identified, increasing the chance of developing a candidate with the potential to enter routine clinical practice. There are two essential requirements that an ideal ^18^F-SSA radioconjugate would fulfil: 1) straightforward radiolabelling procedure that could be automated to yield multi-patient doses in as few radiosynthetic steps as possible; 2) favourable physicochemical and PK properties leading to high tumour uptake and low background noise, i.e., image contrast. Our strategic aim was to employ a ^18^F-prosthetic group strategy with a structure to minimally impact overall lipophilicity of the peptide so that PK properties were derived solely from the structure of the linker group. This would allow more control over fine-tuning the PK properties of the radioconjugate by investigating the linker strategy. In previous examples where lipophilic ^18^F-prosthetic groups were used, the structure of the linker is beholden to correcting the unfavourable physicochemical properties of the prosthetic group. Furthermore, it was anticipated that a simple linker strategy would negate the synthetically challenging and costly precursor synthesis required to incorporate carbohydrate moieties into the radioconjugate.

With this in mind, a unique [^18^F]FET-TOCA peptide library was developed to establish structure-activity relationships (SARs) and select the most appropriate candidates for clinical translation. We identified [^18^F]FET-βAG-TOCA (**7**) as our lead candidate for clinical translation, a novel ^18^F-SSA radioconjugate with suitable chemical, physicochemical and biological properties. Herein, the process of library design, radiochemistry development, biological evaluation, clinical translation and evaluation in humans, and the future direction for [^18^F]FET-βAG-TOCA is discussed.

## 4. Development of the [^18^F]FET-TOCA Library

Our library of radioconjugates would follow the same generic form as shown in Figure 3 and the structural design of the library is rationalised herein.

### 4.1. Radiolabelling Chemistry

Highly lipophilic appendages (e.g., [^18^F]fluorobenzoyl prosthetic group) were to be avoided but the use of hydrophilic prosthetic groups was uncommon as radiolabelling aromatic moieties with fluorine-18 was well explored chemistry with simple purification methods [18]. It was envisaged that the [^18^F]fluoroethyltriazole moiety, a small and hydrophilic appendage, would minimally influence the PK of the radioconjugate. The appendage is accessed by CuAAC “click” chemistry between 2-[^18^F]fluoroethylazide ([^18^F]FEA) and an alkyne-bearing precursor [37,38]. The CuAAC “click” reaction had been successfully evaluated in the context of PET radiochemistry on numerous occasions [39,40]. The resulting 1,2,3-triazole linkage is stable and a bioisostere of an amide bond, therefore attractive for peptide conjugation where perturbation of hydrophilicity is undesirable [41].

Despite the many advantages of this radiolabelling strategy in terms of chemistry and structural properties, the [^18^F]FEA CuAAC “click” strategy was thought to be a challenging prosthetic group to handle due to its volatility. This was a concern for scaling up to meet the demands of clinical doses at the time; there were no PET radiopharmaceuticals in the clinic that had been radiolabelled using CuAAC “click” chemistry and therefore the suitability of this radiolabelling method to clinical translation was unknown. Selecting a radiolabelling strategy that was not yet exemplified in the clinic was a risk, but as the radiochemistry satisfied so many of the design criteria (i.e., hydrophilic, facile radiochemistry) it was worth pursuing. Furthermore, this approach would allow us to exemplify the utility of CuAAC “click” for progressing clinically relevant probes.

### 4.2. Peptide Sequence

Low nanomolar affinity and high specificity towards the SSTR2 subtype of the five SSTR receptors (SSTR1–5) was essential as SSTR2 expression is upregulated in NETs. The peptide [Tyr^3^]octreotate (TOCA) had previously been radiolabelled with a variety of radioisotopes and was selected for radiolabelling with fluorine-18 due to its superior properties to octreotide; in comparison, TOCA has a longer biological half-life and higher affinity for the somatostatin receptor [42,43,44]. TOCA derivatives bearing modification to the N-terminus did not show diminished binding affinity therefore providing an ideal reactive handle from which to attach a linker group.

### 4.3. Linker Group

As discussed previously, the lipophilicity of the peptide library was an influential factor in the design of ^18^F-SSA probes; hydrophilic peptides are less likely to exhibit non-specific binding and poor clearance from excretory organs (e.g., liver) which can result in poor PET image contrast. Many ^18^F-SSA peptides included carbohydrate moieties [19,20,27,28,29,30,45], aspartic acid residues [19] or quaternary amines [22] as part of an elaborate linker moiety between the ^18^F-prosthetic group and the octreotate peptide to maintain hydrophilicity. Although effective, elaborate linker moieties increase the complexity of radiochemistry precursor synthesis and therefore are likely to increase production costs; high production costs may be an influential factor on deciding which radioconjugate to take into routine practice. To maintain hydrophilicity and avoid synthetic complexity, we explored polyethyleneglycol-6 (PEG-6) linkers and amino acid linkers (e.g., glycine, G and β-alanine, βA) and synthesised a library of non-radioactive and radioactive ^18^F-SSA candidates for evaluation.

### 4.4. The Library

The structure-activity relationships (SAR) of the [^18^F]FET-TOCA library were established by modifying one structural variable and determining its influence on biological outcomes; from here, promising candidates were selected for further radiochemistry development. Using the criteria discussed, a focused library of fluorine-containing [Tyr^3^]octreotate were synthesised (Table 1, compounds 7–11) [33]. All compounds contained the fluoroethyltriazole moiety and it was anticipated that the fluorine-18 radiolabelled derivatives would be accessible using similar reaction conditions. The peptide sequence remained identical in all five candidates, with only modification to the linker group in each case. Three candidates contained short amino acid sequences, [^18^F]FET-βAG-TOCA (**7**), [^18^F]FET-G-TOCA (**10**), and [^18^F]FETE-TOCA (11). A negative control for [^18^F]FET-βAG-TOCA (**7**) was synthesised ([^18^F]FET-βAG-[W-c-(CTFTYC)K], 7*) by scrambling the peptide sequence to inhibit SSTR2 binding. Two compounds contained PEG moieties ([^18^F]FETE-PEG-TOCA, **8** and [^18^F]FET-G-PEG-TOCA, **9**), differing only by the terminal amino acid in the linker; this structural moiety is often used to aid the hydrophilicity and solubility of lipophilic compounds. Three compounds, [^18^F]FET-βAG-TOCA (**7**), [^18^F]FET-G-TOCA (**10**), and [^18^F]FETE-TOCA (**11**) contained different amino acid linkers. The library of ^19^F-SSA conjugates and radiochemistry precursors were synthesised and radiolabelling methodology developed for biological evaluation.

## 5. Radiochemistry for Pre-Clinical Studies

**Scheme 1:**
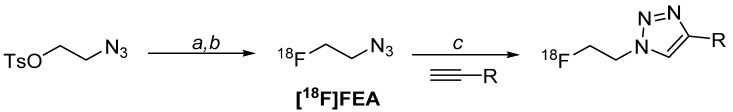
Generic CuAAC “click” radiolabelling method. Reaction conditions: (**a**) K_222_, KHCO_3_, MeCN, 80 °C, 5 min; (**b**) purification by distillation in acetonitrile; (**c**) CuSO_4_.5H_2_O, Na-ascorbate, BPDS, MeCN, H_2_O, 10 min, r.t. [33,46].

The [^18^F]FET-TOCA library was radiolabelled in two steps (Scheme 1): firstly, [^18^F]FEA was synthesised from the 2-azidoethyl 4-methylbenzenesulfonate (**12**) precursor. After purification of [^18^F]FEA by distillation, it was conjugated to the desired propanyl-TOCA precursor via CuAAC “click” chemistry. The resulting radioconjugate was purified by semi-preparative HPLC and the radioactive product reformulated into biologically compatible media by solid-phase extraction (SPE) cartridge. The radiolabelling process was compatible with all candidates in the [^18^F]FET-TOCA library.

One of the most challenging aspects of the radiosynthesis was the purification of the [^18^F]FEA prosthetic group. [^18^F]FEA is volatile and at the time of developing the radiochemistry for the [^18^F]FET-TOCA library, could only be purified by distillation in acetonitrile. The distillation was not ideal due to concerns of potential release of volatile radioactivity into the environment and possible breach of local radiation safety and environmental regulations. The automated radiosynthesis of [^18^F]FEA and subsequent distillation into an external reactor was developed on the GE FASTLab™ automated platform; typically, ca. 10% of the starting radioactivity was lost as volatile radioactive compounds. The [^18^F]FEA prosthetic group in acetonitrile was then used in manual radiosynthesis steps to investigate CuAAC “click” reaction conditions.

The CuAAC “click” chemistry is a well-established method for the formation of 1,2,3-triazole linkage in organic chemistry, but it has become an increasingly popular reaction for radiolabelling molecules with PET radionuclides, as well as incorporating radioisotopes of iodine (e.g., ^123^I, ^131^I, ^125^I, ^124^I) for single-photon emission computed tomography (SPECT), PET and radionuclide therapy [47,48]. Although many catalytic systems are reported, only a small number of popular systems were investigated. The combination of CuSO_4_ and sodium ascorbate, where sodium ascorbate reduces Cu(II) into the active Cu(I) species in situ, proved efficient in accessing the [^18^F]FET-TOCA library. Reaction rates were improved by the addition of bathophenanthrolinedisulfonic acid disodium salt (BPDS), a ligand to stabilize the active Cu(I) species [49,50,51]. Reaction conditions were optimised to improve analytical yields for all [^18^F]FET-TOCA radioconjugates, including the molar equivalences of CuSO_4_, sodium ascorbate, BPDS and reaction temperature.

All peptides in the [^18^F]FET-TOCA library were successfully radiolabelled in a part-automated two-step radiosynthesis using these reaction conditions, although with some variability in decay-corrected radiochemical yield (RCY, ranging from 40%–64%). Variation in radiolabelling efficiency was a direct result of the linker used. Radioconjugates were produced in >98% radiochemical purity in a total synthesis time of approximately 1.5 h. Radiolabelling the [^18^F]FET-TOCA library allowed for the influence of linker strategy on LogD to be evaluated; all peptides were hydrophilic with LogD ranging between −1.5 and −2.77, with [^18^F]FET-G-PEG-TOCA, [^18^F]FETE-PEG-TOCA and [^18^F]FET-βAG-TOCA among the most hydrophilic. With access to the [^18^F]FET-TOCA library in excellent RCY, in vitro and in vivo biological properties were determined.

## 6. Biological Evaluation and Candidate Selection

**Figure 4:**
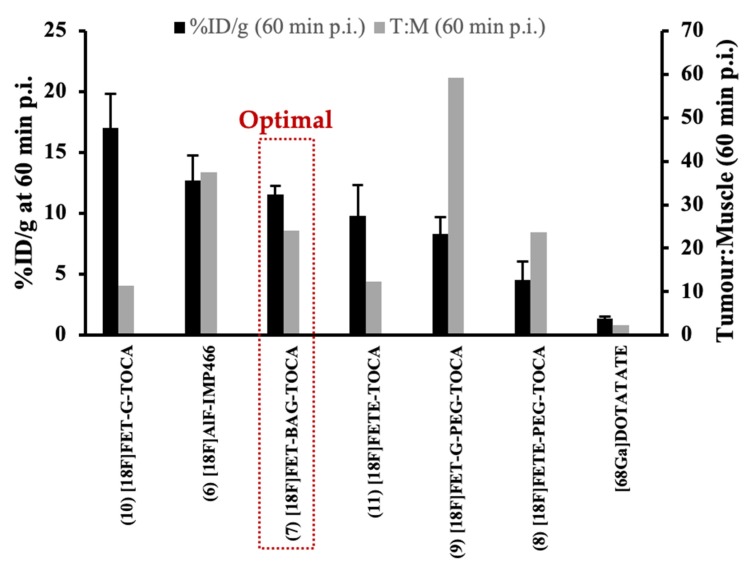
Tumour uptake (%ID/g) and tumour:muscle ratio at 60 min p.i. of the [^18^F]FET-TOCA library in mice bearing AR42J xenografts.

The ideal characteristics of an ^18^F-SSA are high tumour uptake with low non-target accumulation and rapid elimination. The [^18^F]FET-TOCA library was evaluated in vitro and in vivo to select an optimal radioconjugate for PET imaging of SSTR2 positive tumours [35]. The library exhibited high binding affinity ranging from 4–19 nM for SSTR2 and low SSTR4 affinity (6.5–>10 mM). The evaluation in vivo confirmed rapid elimination from non-target tissue and produced high-contrast PET images, but also showed that the structural differences in linker moiety directly influenced uptake in tumour and non-specific tissues, confirming that linker structure influenced PK of the radioconjugate. The uptake of the [^18^F]FET-TOCA library into key tissues was evaluated in mice bearing high SSTR2-expressing AR42J and low SSTR2-expressing HCT116 tumour xenografts. [^18^F]FET-G-TOCA (10) showed the highest tumour uptake (17.05% ± 2.77% ID/g at 60 min p.i.) and [^18^F]FETE-PEG-TOCA (8) showed the lowest tumour uptake (4.51% ± 1.52% ID/g at 60 min p.i.) in AR42J xenografts. Despite the highest tumour uptake of 8, specificity was poor resulting in a tumour-to-muscle (T:M) ratio (60 min) of 11.44 compared to that of [^18^F]FET-G-PEG-TOCA (9) with 59.21. When tumour uptake and tissue specificity were considered together, [^18^F]FET-βAG-TOCA (7) showed the most favourable properties overall with a tumour uptake of 11.58% ± 0.67% ID/g at 60 min p.i. and T:M ratio (60 min) of 24.12 (Figure 4). Uptake of [^18^F]FET-βAG-TOCA was blocked (3.93% ± 0.99% ID/g at 60 min p.i.) by the pre-injection of unlabelled octreotide (10 mg/kg), and uptake was low (0.52% ± 0.39% ID/g at 60 min p.i.) in mice bearing low SSTR-2 expressing HCT116 xenografts. Specificity was also confirmed by the evaluation of a scrambled derivative of [^18^F]FET-βAG-TOCA, which showed low tumour uptake (0.22% ± 0.12% ID/g at 60 min p.i.) in AR42J xenografts. No radioactive metabolites of [^18^F]FET-βAG-TOCA were found in plasma obtained at 30 min p.i. suggesting excellent metabolic stability of the radioconjugate. The tumour uptake of [^18^F]FET-βAG-TOCA was similar to [^18^F]AlF-IMP466 (6), albeit with a slightly lower T:M ratio (60 min), 24.12 vs. 37.44 respectively. All the [^18^F]FET-TOCA radioconjugates outperformed [^68^Ga]Ga-DOTA-TATE [35].

The lead candidate [^18^F]FET-βAG-TOCA was selected for clinical translation based upon in vitro and in vivo properties. Although it did not exhibit the highest tumour uptake or T:M ratio of compounds evaluated from the library, it was selected based upon its combination of favourable uptake and specificity (Figure 4). It was hoped that the insight gained into the biodistribution, pharmacokinetics and metabolism of [^18^F]FET-βAG-TOCA from the in vivo evaluation would translate similarly into humans.

## 7. Radiochemistry for Clinical Studies

A robust, good manufacturing procedure (GMP) compliant radiosynthesis is essential for translating a radiopharmaceutical into the clinic. Automated radiochemistry using commercially available synthesis modules not only protects production scientists from exposure to large doses of radioactivity, but also assists in the manufacture of GMP radiopharmaceuticals by standardizing chemistry and facilitating batch recording. Commercial suppliers of radiochemistry automation hardware and consumables have honed their devices to handle the relatively simple radiochemistry necessary to produce [^18^F]FDG, owed to its high demand and commercial value (cyclotron facilities typically base their business model around [^18^F]FDG production). Simple molecules that followed a similar synthesis scheme to [^18^F]FDG, in brief, a [^18^F]fluoride drying step followed by S_N_2 displacement of a leaving group, a hydrolysis (acid/base), final purification/reformulation, are easily amenable for these systems. It is rare that a radiopharmaceutical synthesised via a prosthetic group approach is translated into a GMP compliant radiosynthesis and therefore by taking [^18^F]FET-βAG-TOCA into the clinic, we were not only investigating the potential benefits for using ^18^F-SSTR2 to replace [^68^Ga]Ga-DOTA-TATE, but also pioneering the translation of the CuAAC “click” prosthetic group strategies in general.

Translating CuAAC “click” chemistry onto an automated radiosynthesis platform posed several challenges that would be overcome in the development of a first-generation radiosynthesis to produce GMP compliant radiopharmaceutical grade [^18^F]FET-βAG-TOCA for phase I and II clinical evaluation; further refinements to the radiosynthesis were made in a second-generation methodology. The automated radiosynthesis procedures were developed using the cassette-based GE FASTLab™ platform. Single use cassette-based automated radiosynthesis platforms are attractive for developing protocols for the production of GMP compliant radiopharmaceuticals because they do not require a validated cleaning procedure; furthermore, thinking beyond early-stage clinical trials and towards progressing radiopharmaceuticals into routine clinical practice, a cassette-based radiosynthesis is more convenient for industrial partners to commercialize into “kit-like” products for national and international dissemination.

An agreement with a commercial custom synthesis manufacturer to supply high quality propanyl-βAG-TOCA precursor and fluorine-19 reference material was an essential component in taking [^18^F]FET-βAG-TOCA into the clinic. A reliable source of precursor and reference standard meant that trials could progress with ease, and scanning opportunities were not missed due to precursor availability. The propanyl-βAG-TOCA precursor was synthesised commercially on contract by ABX advanced biochemical compounds GmbH (Radeberg, Germany) to a good technical grade; the final purification step in the radiosynthesis of [^18^F]FET-βAG-TOCA precluded the necessity of a fully certified GMP precursor for phase I and II clinical studies. A supplier of GMP grade 2-azidoethyl 4-methylbenzenesulfonate precursor was also sourced (Onyx Scientific Ltd., Sunderland, UK).

### 7.1. Development of the First-Generation Radiosynthesis of [^18^F]FET-βAG-TOCA

**Figure 5:**
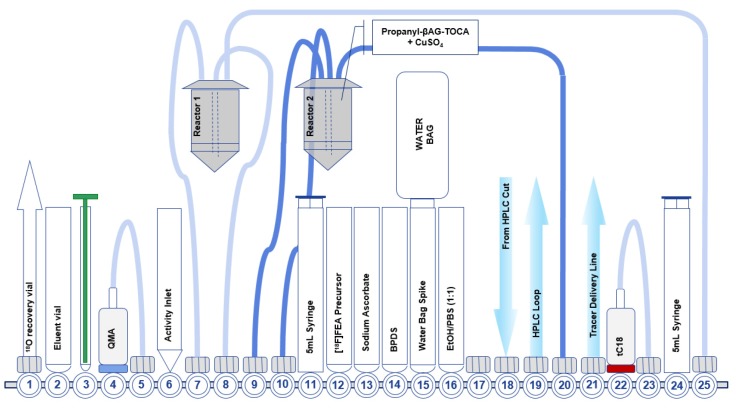
First generation FASTLab™ cassette developed for the GMP radiosynthesis of [^18^F]FET-βAG-TOCA.

The first-generation radiosynthesis of [^18^F]FET-βAG-TOCA included the purification of [^18^F]FEA by distillation. When developing the automated radiosynthesis for early clinical trials, distillation was the only purification method for [^18^F]FEA. The [^18^F]FEA was synthesised and distilled into an off-board Wheaton vial containing the propanyl-βAG-TOCA (Figure 5). It was not possible to include any active cooling apparatus on the distillation setup so condensation of [^18^F]FEA in acetonitrile was purely passive at ambient temperature. Although the distillation was relatively inefficient and losses were assigned to incomplete distillation and volatile radioactive release, the recovery was more than adequate to support multi-patient doses. It was of vital importance to ensure that the inlet and outlet needles of the off-board Wheaton vial were set up precisely as small deviations in their position resulted in poor [^18^F]FEA recovery. After the distillation, the CuAAC “click” reaction was initiated by the addition of sodium ascorbate into the Wheaton vial. The vial contained the propanyl-BAG-TOCA precursor and copper (II) sulfate pentahydrate (CuSO_4_·5H_2_O) before the start of synthesis; this ensured that the reaction volume remained low to facilitate a high reaction efficiency. The reaction proceeded at room temperature, after which it was purified by semi-preparative HPLC to efficiently separate the propanyl-βAG-TOCA precursor from [^18^F]FET-βAG-TOCA. Due to the relative fragility of the core peptide structure, radiolysis was a concern and indeed, when high starting activities of [^18^F]fluoride were used (>10 GBq), degradation was observed. Clinical production required the starting activity to be limited to 10 GBq, which reduced radiolysis. Ascorbic acid was used throughout the radiosynthesis as a radical scavenger which prevented radiolytic cleavage of the peptide at lower activities; ethanol (1%) was included in the semi-preparative HPLC mobile phase to protect against radiolysis during the purification. The radiosynthesis produced [^18^F]FET-βAG-TOCA in 6.5% ± 2.9% RCY (n.d.c) with a molar activity >150 GBq/μmol [34].

Quality control was an essential part of taking [^18^F]FET-βAG-TOCA into clinical studies as an investigational medicinal product (IMP). The limit of non-radioactive impurities (e.g., BPDS determined by HPLC) per administered dose was set at <12 μg with not greater than 1 μg attributed to the propanyl-βAG-TOCA precursor. No more than 10 μg of [^19^F]FET-βAG-TOCA was allowed per administered dose. The amount of copper salts in the dose formulation, attributed to the CuSO_4_ catalyst used in the CuAAC “click” chemistry, was determined by ion chromatography (IC) in the initial validation of [^18^F]FET-βAG-TOCA and one in 10 clinical doses were assessed routinely. The radiopharmaceutical complied with (European Medicines Agency) EMEA “guideline on the specific limits for residual metal catalysts” with no more than 25 ppm per administered dose.

### 7.2. Development of the Second-Generation Radiosynthesis of [^18^F]FET-βAG-TOCA

In 2018, we investigated new automated methodologies to facilitate CuAAC “click” radiochemistry on the GE FASTLab™ platform [46]. Some years prior, Zhou et al (2015) described an innovative method of purifying [^18^F]FEA by tC18 and HLB SPE cartridges to separate unreacted 2-azidoethyl 4-methylbenzenesulfonate from [^18^F]FEA, avoiding distillation [46]. The combined use of tC18 and HLB SPE cartridges in tandem trapped unreacted precursor on the tC18 SPE cartridge, whilst unretained [^18^F]FEA was subsequently trapped on the HLB SPE cartridge. In addition to purifying [^18^F]FEA, CuAAC “click” reactions were performed on the HLB SPE cartridge if all reagents were carefully loaded in a small reaction solvent volume. With interest in the new approach for purifying [^18^F]FEA, we sought to develop a new automated radiosynthesis of [^18^F]FET-βAG-TOCA to utilize the new technology. A few challenges were anticipated in translating the SPE cartridge based purification onto an automated radiosynthesis platform: firstly, the efficiency of CuAAC “click” chemistry is strongly dependent upon reactant and reagent concentration, however in the context of automated PET radiochemistry, manipulating small reaction volumes is notoriously difficult using commercial automated radiosynthesis platforms due to systematic losses attributed to dead volumes in the cassette, tubing and reagent vials. We anticipated that developing an automated procedure to take advantage of the SPE purification method would not only reduce the release of radioactive volatiles by negating the distillation step, but also eliminate the requirement for an off-board reactor for the CuAAC “click” reaction between [^18^F]FEA and the propanyl-βAG-TOCA precursor and allow for small reaction volumes to be contained within the HLB SPE cartridge and promote efficient radiolabelling of [^18^F]FET-βAG-TOCA.

**Figure 6:**
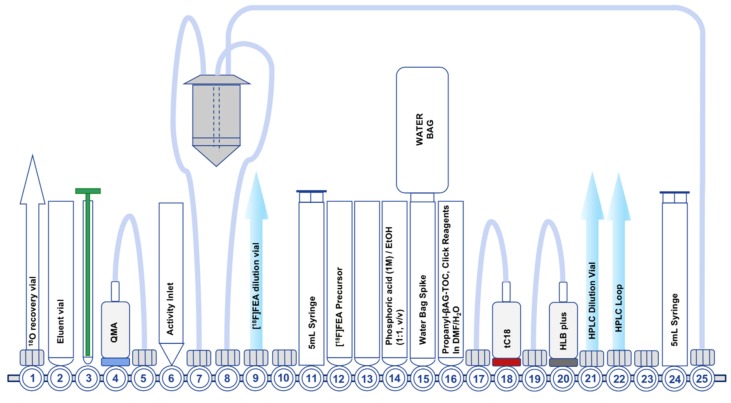
Second generation FASTLab™ cassette developed for the radiosynthesis of [^18^F]FET-βAG-TOCA.

The second-generation radiosynthesis of [^18^F]FET-βAG-TOCA took advantage of the SPE purification of [^18^F]FEA and subsequent on-cartridge CuAAC “click” reaction (Figure 6); the radiolabelling proceeded efficiently with an isolated radiochemical yield (non-decay corrected) of 16.7% ± 0.6% [46]. We noticed a 10-fold reduction (1% of the starting activity) in the quantity of radioactivity released into the environment compared to the first-generation radiosynthesis. Due to radiation safety limitations in our preclinical research laboratory, we were unable to use high starting activities of [^18^F]fluoride (>6 GBq) to examine if concentrating the CuAAC “click” reaction into a small volume inside the HLB SPE cartridge would accentuate radiolysis more than the first-generation radiosynthesis; at our current starting activities (ca. 6 GBq) we did not see radiolytic degradation of [^18^F]FET-βAG-TOCA.

Due to limited space on the FASTLab™ cassette, we were unable to include an SPE reformulation step, which led to the development of biocompatible preparative HPLC solvent systems based around ethanol and phosphate buffer (pH 2.4). Unfortunately, using these conditions we were unable to efficiently separate [^18^F]FET-βAG-TOCA from the propanyl-βAG-TOCA precursor, resulting in poor molar activity (A_m_ = 0.5 GBq/µmol) [46]. Our pre-clinical studies using [^18^F]FET-βAG-TOCA are supported by the second-generation radiosynthesis in combination with the preparative HPLC purification solvent system as described for the first-generation radiosynthesis; for these studies, we can reformulate the “cut-peak” manually into biocompatible solvents. Optimization of the cassette design to either include a formulation step, or take the formulation step off-board, is ongoing and we anticipate that the second-generation radiosynthesis will support clinical trials in the future. It should be possible to translate either the first or second generation radiosynthesis method onto other automated radiosynthesis platforms other than the GE FASTLab™. Implementing a solid-supported azide reagent to sequester unreacted propanyl-βAG-TOCA may also provide a useful strategy to improve molar activity of [^18^F]FET-βAG-TOCA [52].

We have radiolabelled libraries of alkyne containing small-molecule and peptides (data not published) with [^18^F]FEA using the second-generation radiosynthesis, demonstrating the method to be robust and generally applicable other substrates.

## 8. Additional Pre-Clinical Evaluation to Support First-in-Human Use of [^18^F]FET-βAG-TOCA

Two key studies were conducted to assure regulators of the safety of [^18^F]FET-βAG-TOCA prior to first-in-human testing. A Good Laboratory Practice single dose intravenous bolus extended acute toxicology study in the rat (HsdHan^TM^:WIST strain) was conducted with both [^19^F]FET-βAG-TOCA and the propanyl-βAG-TOCA precursor. Animals were monitored throughout; day 2 (interim) and day 14 (termination) analysis were conducted. Dose levels of 0, 0.25 and 1 mg/kg of either test articles were tolerated in-life with no mortality. There were no effects on food consumption or body weight. Clinical pathology changes were noted but were not consistently seen in both sexes and were not associated with any pathological changes. A No Observed Adverse Effect Level (NOAEL) for both [^19^F]FET-βAG-TOCA and propanyl-βAG-TOCA was considered to be 1.0 mg/kg. This study followed ‘approach 1’ given in the ICH M3 (R2) guideline to assess whether a single 50 μg (up to 100 μg) dose of the test article can be given to humans. The study design permitted a safety margin of 1000× the equivalent human dose to be established.

While adhering to ALARP principles (As Low As Reasonably Practical), a rat dosimetry study was also conducted to enable initial prediction of human Effective Dose. Wistar rats were dosed with a single intravenous injection of [^18^F]FET-βAG-TOCA and monitored up to 7 h. Effective Dose for both sexes were estimated at 0.03 mSv/MBq.

## 9. Clinical Evaluation

The first-in-human clinical translation of [^18^F]FET-βAG-TOCA, was conducted in locally advanced and metastatic NETs [34]. This study was designed in two phases; phase 1 assessing safety, biodistribution and dosimetry and; phase 2, assessing tumoural uptake and a comparison of [^18^F]FET-βAG-TOCA and [^68^Ga]Ga-DOTA-TATE imaging. In Phase 1, [^18^F]FET-βAG-TOCA was found to be safe, with no differences in biodistribution between male and female subjects. Little or no defluorination was observed and physiological uptake was noted in pituitary, salivary glands and thyroid tissue. In addition, [^18^F]FET-βAG-TOCA showed high tumoural uptake, as well as high tumour/background contrast in all organs including the liver (a key site for metastases in NETs). Metabolite analysis showed that over 60% of parent radioligand was detectable in plasma at 60 min and over 30% at 2.5 h, demonstrating potential use in late-imaging protocols, in clinical practice. Overall [^18^F]FET-βAG-TOCA was found to be rapidly eliminated from most organs leading to relatively low residence times and low/stable organ radioactivity within 60 min of post-injection. The main pharmacokinetic difference observed between [^18^F]FET-βAG-TOCA and ^68^Ga-radiolabelled somatostatin analogues, was the highest absorbed dose received by source organs. Due to primarily hepato-biliary and renal excretion of [^18^F]FET-βAG-TOCA, highest absorbed dose was seen in the gallbladder, followed by the spleen, stomach wall, liver, kidneys and bladder; this finding together with high uptake in tumours perhaps limits false positive detection due to hepatobiliary elimination. This is in contrast to ^68^Ga-radiolabelled analogues, which have predominantly renal elimination [53,54,55]. Dosimetry of [^18^F]FET-βAG-TOCA was similar to other ^18^F-radiolabelled somatostatin analogues, with mean ED 0.029 ± 0.004 mSv/MBq, closely predicted by the rat studies. The promising results of phase 1, and now phase 2 (to be published), highlight the enormous potential of this radioligand in the therapeutic decision making of NET patients and also for future SSTR2-imaging studies.

## 10. Comparison of ^18^F-Radiolabelled Octreotide Analogues

Of the fluorine-18 radiolabelled octreotide analogues shown in Table 1, four radioconjugates including [^18^F]FET-βAG-TOCA have been evaluated in humans (entry 1, 4, 6 and 7). Single-step radiolabelling protocols to produce 4 and 5 are attractive and result in excellent RCY, however only manual radiosynthesis methods have been described. The radiosynthesis of 4 requires precise manipulation of small reaction volumes and would benefit from the development of an automated procedure to produce the radioconjugate on scale [56]. [^18^F]FP-Gluc-TOCA (1) is the most complex radioconjugate to produce with a total of five radiosynthetic steps and two HPLC purifications, which is not compatible with routine production to satisfy the growing demand for SSTR2 stratification, despite excellent preclinical and clinical performance. The [^18^F]AlF-NOTA-octreotide (6) and [^18^F]FET-βAG-TOCA (7) radioconjugates are synthesised in two synthetic steps, albeit the rapid metal-based [^18^F]AlF radiolabelling of 6 achieves the desired radioconjugate 50% faster than CuAAC “click” radiolabelled [^18^F]FET-βAG-TOCA, which is one contributing factor to the superior RCY of 6 [31,34,36]. The uptake of [^18^F]FET-βAG-TOCA and [^18^F]AlF-NOTA-octreotide in AR42J tumour models is similar (ca. 12% ID/g at 60 min p.i.). Both radioconjugates are being evaluated in the clinic in a head-to-head study with [^68^Ga]Ga-DOTA-TATE [32]. The GMP compliant automated radiosynthesis of [^18^F]AlF-NOTA-octreotide effectively produces the radioconjugate on a large scale (9–12 GBq) in a high RCY of 26% (decay corrected) within 40 min when starting from large activities of [^18^F]fluoride (44 -64 GBq) [31]. The RCY for the first-generation radiosynthesis of [^18^F]FET-βAG-TOCA was ca. 12% (decay corrected). Improvements made in the second-generation automated method boosted the yield to 23% (decay corrected) within 75 min [46]; further development to produce clinical doses on a large scale is ongoing.

## 11. Lessons for the Clinical Translation of ^18^F-Radiolabelled Peptides

The bench-to-bedside journey of [^18^F]FET-βAG-TOCA may be used to demonstrate a clear and logical strategy for taking other fluorine-18 radiolabelled peptides into the clinic. Important considerations learned from this work which may be applicable to other projects include:

- Defining clear aims of what a successful ^18^F-peptide will achieve, in terms of radiolabelling chemistry, biological properties and clinical translation.
- Implement a focused library with appropriate candidates and a generic radiolabelling method that can be applied to all, which ultimately simplifies their biological evaluation.
- Develop chemistry that supports clinical translation, but does not stop innovation when phase I and II trials have been completed; look to the future, asking whether the current method is adequate for taking this compound forwards into routine clinical practice? Are there better radiolabelling methods available which may alleviate challenges or improve production yield, that were not available for the first GMP compliant radiosynthesis?

## 12. Conclusions

The development of [^18^F]FET-βAG-TOCA from bench-to-bedside is a testament to the multidisciplinary of our research group and our researchers who have championed translational science. The reach of [^18^F]FET-βAG-TOCA is wide and far, not only in its progression into phase III clinical trials, but as it continues to inspire new research projects in our group and exemplifies the possibilities for automating challenging radiochemistry for clinical grade radiopharmaceuticals. Radiochemistry advancements resulting from but, collateral to [^18^F]FET-βAG-TOCA, advocates the importance of establishing generic “toolbox” radiolabelling procedures for classes of compounds/functional groups, so that radiolabelled compounds of interest can be accessed quickly with a clear route to the clinic; we encourage the inclusion of the [^18^F]fluoroethyltriazole moiety in our library designs for future compounds as we know the potential to quickly progress candidates into the clinic now that we have robust radiolabelling methodology established in-house. We hope that describing the discovery and translational journey of [^18^F]FET-βAG-TOCA, from library design, pre-clinical and translational radiochemistry, biological evaluation and first-in-human trial, informs, encourages and inspires others to embark on their journey, moving radiopharmaceuticals from the bench into patients and, ultimately, impacting the lives of cancer patients worldwide.

## Figures and Tables

**Table 1:** Summary of ^18^F-SSA radioconjugates discussed in this review.

Entry.		Compounds	Synthesis Time (min)	Synthetic Steps	Automated synthesis?	GMP Compliant?	Evaluated in Humans?											Ref
								RCP (%)	RCY (%)	A_m_ (GBq/μmol)	SSTR2 IC_50_ (nM)	Model	LogD	Tumour (%ID/g at 60 min p.i.)	Tumour:Bone (1 h)	Tumour:Muscle (1 h)	Tumour:Liver (1 h)	
**Published ^18^F-SSTRadioconjugates**	**1**	[^18^F]FP-Gluc-TOCA	180	5	ND	Y	Y	>98	20–30	>37	2.8 ± 0.4	AR42J	ND	13.54 ± 1.47	20	56	19	[26,27]
	**2**	Cel-S-Dpr([^18^F]FBOA)TOCA	50	2	ND	N	N	>98	40–60	ND	1.8 ± 1.1	AR42J	ND	24.04 ± 2.50	28	200	27	[28,29]
	**3**	[^18^F]FGlc-TATE	70	3	Y	N	N	>98	19–22	32–106	4.2	AR42J	ND	5.62 ± 1.53	9.7	12.2	6.2	[30]
	**4**	[^18^F]SiFA*lin*-Glc-Asp _2_ -PEG_1_-TATE ([^18^F]SiFA*lin*-TATE)	25	1	ND	Y	Y	>98	~50	44–63	14.4 ± 1.2	AR42J	ND	18.51 ± 4.89	ND	39.35	8.97	[19,20,24]
	**5**	[^18^F]AMBF_3_-TATE	25	1	ND	N	N	>99	>30	>111	0.13 ± 0.03	AR42J	ND	10.11 ± 1.67	21.3	92	26.2	[21,22]
	**6**	[^18^F]AlF-NOTA-octreotide ([^18^F]AlF-IMP466)	45	2	Y	Y	Y	>98	~50	36.1	3.6 ± 0.6	AR42J	ND	12.73 ± 2.05	86 (2h)	37.44	ND	[23,31,32,36]
**[^18^F]FET-TOCA analogues**	**7**	[^18^F]FET-βAG-TOCA	90	2	Y	Y	Y	>98	~6.5 66^dc^	224–562	1.6 ± 0.2	AR42J	−2.26	11.58 ± 0.67	ND	24.12	ND	[33,34,35]
	**7**	[^18^F]FET-βAG-TOCA										HCT116		0.52 ± 0.39	ND	0.97	ND	[33,34,35]
	7*	[^18^F]FET-βAG-[W-c-(CTFTYC)K]	90	2	Y	N	N	>98	64^dc^	12.3	> 10.0 mM	AR42J	−1.14	0.22 ± 0.12	ND	0.65	ND	[33,35]
	**8**	[^18^F]FETE-PEG-TOCA	90	2	Y	N	N	>98	52^dc^	5.9	13.2 ± 7.8	AR42J	−2.77	4.51 ± 1.52	ND	23.74	ND	[33,35]
	**9**	[^18^F]FET-G-PEG-TOCA	90	2	Y	N	N	>98	40^dc^	4.8	10.8 ± 5.9	AR42J	−2.68	8.29 ± 1.42	ND	59.21	ND	[33,35]
	**10**	[^18^F]FET-G-TOCA	90	2	Y	N	N	>98	50^dc^	5.9	4.0 ± 1.4	AR42J	−1.83	17.05 ± 2.77	ND	11.44	ND	[33,35]
	**11**	[^18^F]FETE-TOCA	90	2	Y	N	N	>98	51^dc^	8.4	2.9 ± 1.3	AR42J	−1.5	9.79 ± 2.57	ND	12.39	ND	[33,35]
		[^68^Ga]Ga-DOTA-TATE	--	1	--	N	N	>98	ND	ND	14.7 ± 7.7	AR42J	N/A	1.37 ± 0.16	ND	2.28	ND	--

RCP = radiochemical purity, A_m_ = molar activity, ND = not disclosed, Ref = reference. dc = decay corrected.
